# Data Collection Strategy Based on OSELM and Gray Wolf Optimization Algorithm for Wireless Sensor Networks

**DOI:** 10.1155/2022/4489436

**Published:** 2022-02-08

**Authors:** Yang Bai, Li Cao, Shuxin Wang, Haihua Ding, Yinggao Yue

**Affiliations:** School of Intelligent Manufacturing and Electronic Engineering, Wenzhou University of Technology, Wenzhou, 325035, China

## Abstract

In order to effectively reduce the energy consumption, improve the efficiency of data collection in HWSNs, and prolong the lifetime of the overall network, the clustering method is one of the most effective methods in the data collection methods for HWSNs. The data collection strategy of HWSNs based on the clustering method mainly includes three stages: (1) selecting the appropriate cluster head, (2) forming between clusters, and (3) transferring data between clusters. Among them, the selection of the cluster heads in the first stage. The optimal number of cluster heads in the formation of clusters in the second stage is the core and key to the clustering data collection of HWSNs. In the stage of cluster head selection, a data collection strategy for HWSNs based on the clustering method is proposed. Sink establishes an extreme learning machine neural network model. The cluster member nodes select cluster heads based on the remaining energy of the sensor node, the number of the neighbor node, and the distance to the sink. The best cluster head node is selected through the adaptive learning of the online sequence extreme learning machine. Through comprehensive consideration of various factors to complete the clustering process, the gray wolf algorithm is used to optimize the number of clusters, balance the effect of clustering, and improve the efficiency of data collection while reducing energy consumption. An energy efficient and reliable clustering data collection strategy for HWSNs based on the online sequence extreme learning machine and the gray wolf optimization algorithm is proposed in this paper. The simulation results show that the proposed algorithm not only significantly improves the efficiency of the data collection and reduces energy consumption but also comprehensively improves the reliability of the network and prolongs the network's lifetime.

## 1. Introduction

The biggest feature of a heterogeneous sensor network is data collection. First, the source node collects the data and completes the data forwarding through the routing node. The data is transmitted between nodes by wireless communication. The cluster head node performs data fusion and sends it to the sink node [1, 2]. And through the Internet, the data are transferred to the database for analysis and processing, and finally, feedback is given to the user. This process is a series of processing on the collected data. But, for the heterogeneous sensor nodes, the perception ability is limited; the computing ability is limited; and the energy is limited [3]. How to design a high-efficiency data collection strategy, reduce the energy consumption, and improve the efficiency of data collection is currently a key research topic in the field of HWSNs. According to whether the structure of the heterogeneous sensor node data collection process has a hierarchical structure, it can be divided into two data collection structures: a hierarchical cluster structure and a flat structure [4]. The data collection of the flat structure is mainly reflected in the common nodes, routing nodes, and sink nodes in a plane; data collection of the hierarchical structure will cause data transmission congestion, low efficiency, and fewer applications [5]. The data collection mode of hierarchical and clustered structure is much more efficient than the data collection method of flat structure in terms of sensor node organization and management, work efficiency, and network scalability. This clustering structure is particularly suitable for large-scale distributed heterogeneous sensor networks. Therefore, the research on the data collection method of heterogeneous sensor networks with this hierarchical and clustered data collection structure has very important significance [6].

The clustering data collection process of HWSNs is affected by the limited initial energy, perception ability, communication bandwidth resources of heterogeneous nodes, and the dynamic change of the network topology. These restrictive conditions determine that there must be a clustering routing protocol suitable for its own characteristics in the design process of HWSNs clustered data collection [7]. At the same time, in the process of designing the HWSNs clustered data collection protocol, consideration was given to saving energy consumption, improving the efficiency of the data collection, and prolonging the network's lifetime. The cluster routing protocol of HWSNs mainly responds to the dynamically changing network structure. The establishment, maintenance, and smooth transmission of data between sensor nodes and cluster head nodes and sink nodes are very important [8]. At the same time, these characteristics also determine the design of an efficient and reliable cluster routing protocol that has a very important meaning. The clustered data collection routing protocol of HWSNs designed in this paper mainly selects the network layer routing protocol for in-depth research, and the data collection strategy is researched and improved [9].

To achieve the optimal network performance, this paper defines the optimal network performance, that is, the optimization goal, as the problem of minimizing network energy consumption. Through the obtained optimal node awareness rate and physical link transmission rate, the goal of minimizing network energy consumption can be achieved. Combining gray wolf optimization algorithm and online sequence extreme learning machine, the two methods cooperate with each other to optimize the performance of heterogeneous wireless sensor networks, prolong the network's lifetime, and effectively balance the communication energy consumption of the sensor nodes and the collection delay of data collection. Therefore, the purpose of this paper is to propose an efficient and low-energy data collection and network energy-saving optimization strategy so that the network can efficiently collect sensor node data and reduce network energy consumption.

In this work, a new method of data collection strategy for HWSNs based on the online sequence extreme learning machine and the gray wolf optimization algorithm is proposed. In comparison with the current general selection approaches, the main contributions of our work in this paper can be summarized as follows:

1. Characterize the issues of a data collection strategy for HWSNs and establish a mathematical model of data collection strategy for HWSNs.
2. Present a novel data collection strategy based on online sequence extreme learning machine and gray wolf optimization algorithm.
3. Provide extensive simulation results to demonstrate the use and efficiency of the proposed data collection algorithm.
4. Evaluate the performance of the proposed algorithms by comparing them with the coverage optimization algorithms of the SEP, ELM, and ELM + PSO algorithm. The experimental results show that the scheme and strategy proposed in this paper not only ensure the continuous operation of the network but also achieve better network performance.

The rest of the paper is organized as follows: Section 2 discusses the related work.  Section 3 equates the problem of the system model and problem description for the HWSNs.  Section 4 describes the algorithm of the online sequence extreme learning machine and the gray wolf optimization algorithm.  Section 5 presents the applied mathematical models and implementation steps of the clustering data collection algorithm for HWSNs.  Section 6 provides the parameters and simulation results that validate the performance of our algorithm.  Section 7 concludes the paper.

## 2. Related Work

The characteristic of a heterogeneous wireless sensor network is a data-centric network. Data collection is one of the most basic tasks of HWSNs, and it is also a prerequisite for application in various other fields and scenarios. Through research, it is found that the data collection method based on the cluster structure is suitable for the monitoring environments that require large-scale sensor nodes and can improve network load balance and the efficiency of data collection [10]. In the data collection strategy of HWSNs, the data collection methods are classified according to the network structure and working characteristics of the cluster head nodes and the cluster member nodes, which are mainly divided into the following three types.

### 2.1. Cluster-Based Data Collection

According to the characteristics of the sensor node, the appropriate node is selected as the cluster head, and the surrounding cluster member nodes are chosen to join the corresponding cluster according to the actual situation. The routing node transmits information, and the cluster head node performs data fusion and sends it to the sink. Generally, clustering data collection is the most adopted strategy for HWSNs data collection. Dutt et al. [11] proposed a two-layer channel selection threshold method for the problems of large energy consumption and high system complexity in the process of selecting cluster head nodes. Cluster heads are selected by setting appropriate thresholds. This method is referred to as the CREEP clustering data collection strategy. This strategy significantly improves the collection efficiency of the network and improves the quality of service of the network. However, the proposed method increases network energy consumption and algorithm processing time. Verma et al. [12] considered the remaining energy of heterogeneous sensor nodes, calculated the distance between ordinary nodes and cluster heads, and constructed the fitness function according to the network density function. A genetic algorithm-based optimized clustering protocol (GAOC) is proposed, which greatly improves network collection efficiency and reduces network energy consumption, this method increases the processing time of the algorithm and at the same time increases the computational complexity of the algorithm. Al-Kiyumi et al. [10] proposed a distributed energy-aware fuzzy logic routing algorithm (DEFL), which simultaneously solves the problems of energy efficiency and energy balance. The above-mentioned methods reduce the energy consumption of the network and prolong the life of the network, but these methods increase the processing time of the algorithm and at the same time increase the computational complexity of the algorithm.

### 2.2. Tree-Based Data Collection

Tree-based data collection means that the sensor nodes of HWSNs form a tree structure through reasonable combination, and all nodes are divided into child nodes and parent nodes. The role of the child node is to collect and forward data information, and the role of the parent node is to receive data and merge it, thereby improving the efficiency of data collection. In order to reduce the cost of data collection, Sha et al. [13] proposed an energy-balanced data collection method (ETDC) for the data collection on subtrees with similar structures, which can optimize network performance and improve the efficiency of data collection and the reliability of the network. Since the selection of intersection points in route design has an important impact on the timely collection of network data, Nitesh et al. [14] proposed an effective intersection point collection algorithm. The intersection point collection strategy of this method mainly refers to the multipath of data transmission and network energy consumption to generate trees, which greatly improves the efficiency of data collection and work. Gowda et al. [15] considered factors such as threshold, connection time, coverage, and robustness and proposed a link-aware tree data collection method (VEELCT), which greatly improved the efficiency of data collection. The above-mentioned tree-structured data collection method integrates the influence of the remaining energy of the node and the communication cost of the nodes in the cluster on the network survival time and iteratively selects the cluster heads periodically, which effectively avoids the problem of uneven distribution of the cluster heads. However, if the network nodes are not evenly distributed, the load of the nodes will be uneven.

### 2.3. Chain-Based Data Collection

Chain-based data collection means that the sensor nodes in the network are connected in series in a chain to form a data transmission link. The chain structure of data transmission means that in heterogeneous sensor network data collection, heterogeneous nodes form a link for data transmission and select a chain head node, other nodes send data to the chain head, and the chain head forwards the data to the sink. Wang et al. [16] proposed an enhanced energy-efficient collection algorithm EPEGASIS from four aspects to improve the efficiency of data collection in response to the hot issues in the sensor information system. Sasirekha and Swamynathan [17] proposed a cluster chain mobile agent routing algorithm (CCMAR), which improves the collection efficiency and prolongs the life of the network. Qiao and Zhang [18] proposed a random projected polar coordinate chain line (RPC) method. A chain structure network architecture is constructed according to the polar coordinate position, and a random projection method is adopted for data compression and transmission. This method reduces the energy consumption of data collection.

The protocol uses a greedy algorithm to generate a chain. The sensor nodes only need to communicate with their nearest neighbor nodes, which can effectively use energy and greatly improve the network's lifetime. However, the protocol is a chain structure, so the data transmission delays increase, which is not suitable for real-time applications.

Based on the aforementioned documents, it can be summarized as the following three main reasons. (1) The research direction is relatively one-sided. When many researchers study hierarchical cluster routing, if the clustering strategy studied is more complicated, they will neglect the routing between clusters. Conversely, when studying complex intercluster routing, simple and low-performance clustering strategies are used. (2) Clustering or selecting cluster heads has great randomness. Current clustering methods usually determine the candidate cluster heads by determining the thresholds of multiple weight factors of nodes. Then each node randomly generates a random number to select the cluster head with equal probability, which has great randomness. (3) The clustering strategy is one-sided. Many current researches on clustering strategies are based on certain aspects, such as the idea of nonuniform clustering, graph theory, energy perception, and geographic location. These strategies only consider a single aspect, and performance needs to be improved.

Based on the analysis of the above references, the current clustered data collection method unilaterally reduces the collection energy consumption, prolongs the network's lifetime, or improves the efficiency of data collection, without comprehensively considering the network performance and the balance of the energy consumption of the data transmission. Through comprehensive analysis of the above, in this paper, we comprehensively consider the remaining energy of the heterogeneous sensor nodes in the data transmission process, the distance between the heterogeneous sensor nodes and sink, the number of neighbor nodes to complete energy-saving, efficient, and reliable data collection. The clustering data collection of HWSNs is divided into three stages. In the first stage, improper or frequent selection of cluster heads for HWSNs clustering nodes is likely to cause network instability, dynamic changes in the topology, and low data transmission efficiency between heterogeneous nodes. In the cluster head selection stage of HWSNs clustering data collection, an extreme learning machine model is established at the sink node, and the remaining energy of the node, the number of node neighbors, and the distance to the sink are used as input. The best cluster head is selected through the adaptive learning of the online sequence extreme learning machine. The second stage is the clustering stage of HWSNs. The number of clusters affects the efficiency of data collection and energy consumption. A gray wolf optimization algorithm for the optimal number of clusters of HWSNs is designed. In the third section, the data collected by HWSNs heterogeneous nodes are transmitted to the cluster head node, and the cluster head node data is fused and forwarded to the sink. This paper proposes an energy-saving, efficient, and reliable clustering data collection strategy for HWSNs based on the online sequence extreme learning machine and gray wolf optimization algorithm, which reduces the transmission delay of heterogeneous nodes, effectively improves data collection efficiency, and balances network energy consumption.

## 3. System Model and Problem Description

This article first describes the clustering data collection process of HWSNs, constructs a schematic diagram of data collection scenarios, and then establishes a network energy consumption model and an optimized cluster number model.

### 3.1. Clustering Data Collection Model

**Figure 1:**
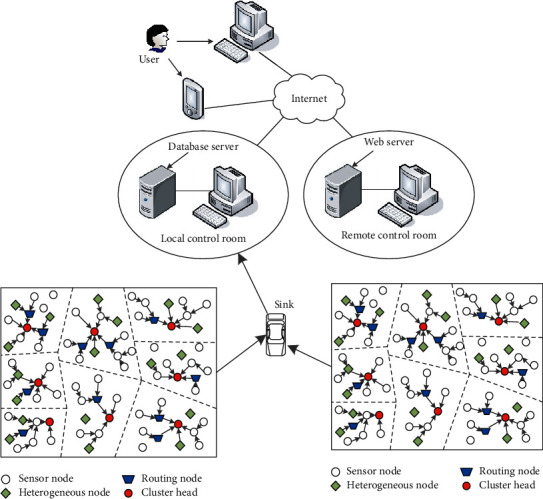
Schematic diagram of clustered data collection of HWSNs.

The clustered data collection method of HWSNs in this paper is based on the improvement and in-depth study of the classic distributed low-power adaptive hierarchical routing protocol (stable election protocol, SEP). In the three stages of the clustered data collection strategy of HWSNs, the first stage selects cluster heads based on the information of the heterogeneous sensor nodes; the surrounding common sensor nodes independently choose to join the corresponding clusters; and the sensor network forms a hierarchical network structure. The information collected by the terminal node is forwarded to the cluster head node through routing and then sent to the sink node after simple data fusion [19]. The schematic diagram of HWSNs clustered data collection is shown in Figure 1.

As shown in Figure 1, heterogeneous nodes and ordinary nodes are randomly deployed in the square monitoring area, and the nodes will not move after the deployment is completed. The fixed sensor node sends the collected data to the sink node, and the sink node in this article is also fixed. Under the premise of the specified transmission delay, the node collects the information and finally sends it to the sink node. First, the cluster heads must be selected, and a suitable clustering structure must be formed [20]. The routing node selects the appropriate path to transmit the data to the cluster head node and then forwards the data to the sink after fusion. The sink uploads the collected data to the upper computer, and the user checks the occurrence information of the monitoring area through the terminal device. Through multiple rounds of data collection, the data collection of the entire heterogeneous wireless sensor network is finally completed.

### 3.2. Optimal Cluster Head Number Model

First, a clustering mathematical model of HWSNs is established based on energy consumption. The monitoring area is a square of length *M*, and the total number of nodes is *N*, including *K* cluster head nodes, and there are *N*/*K*-1 cluster member nodes. The energy consumption generated by the data fusion is *E*_CH_, and then the information is transmitted to sink, and the distance between the cluster head and the base station is *d*_ChtoBS_. The energy consumption of cluster head nodes mainly includes receiving data sent by the heterogeneous sensor nodes and forwarding data to sink. The energy consumption *E*_NEN_ of cluster member nodes includes collecting data and forwarding data to the cluster head [21].

The ordinary sensor nodes and the heterogeneous sensor nodes in the sensing area obey the Poisson distribution; the area of the cluster is *S* = *M*^2^/*k*; and the density function of the nodes in the monitoring area is *ρ*(*x*, *y*), which is calculated as shown in the following formula:

(1) $$\begin{array}{c} \rho \left(x,y\right)=\left\{\begin{array}{cc} \frac{k}{M^{2}}, & \left(x,y\right)\in S,\\ 0, & \left(x,y\right)\notin S. \end{array}\right). \end{array}$$

The energy consumption of the cluster head node to receive data is proportional to the distance. The mathematical expectation value of the square of the distance between the cluster head node and the cluster member nodes is calculated as follows:

(2) $$\begin{array}{c} E\left[d_{\text{toCH}}{}^{2}\right]=E\left[x^{2}+y^{2}\right]=\frac{M^{2}}{2k\pi }. \end{array}$$

The calculation formula for the total energy consumption of all nodes in the heterogeneous sensor network is as follows:

(3) $$\begin{array}{c} E_{\text{total}}=k\times E_{\text{cluster}}=k\times E_{\text{CH}}+k\times \left(\frac{N}{k-1}\right)\times E_{\text{NEN}}, \end{array}$$

(4) $$\begin{array}{c} E_{\text{total}}=k\times E_{\text{cluster}}\\ =k\times \left(\left(\frac{N}{k-1}\right)\times lE_{\text{elec}}+lE_{\text{elec}}+l\varepsilon_{\text{amp}}d_{\text{CHtoBS}}^{2}\right)+\left(N-k\right)\times \left(lE_{\text{elec}}+l\varepsilon_{\text{amp}}d_{\text{toCH}}^{2}\right)\\ =l\left(2NE_{\text{elec}}-kE_{\text{elec}}+k\varepsilon_{\text{amp}}d_{\text{CHtoBS}}^{2}+\left(N-k\right)\times \left(\varepsilon_{\text{amp}}d_{\text{toCH}}^{2}\right)\right). \end{array}$$

In formula (4), only the value of *d*_toCH_ is uncertain. In this calculation formula, since the position and number of cluster heads must change during each polling data collection process, *d*_toCH_ is solved by calculating the mathematical expectation value. The range of each cluster area is a circular area with a radius of $M/\sqrt{\pi K}$. The formula for calculating the expected value of the square of the distance between the heterogeneous node and the cluster head node is as follows:

(5) $$\begin{array}{c} E\left[d_{\text{toCH}}^{2}\right]=\iint \left(x^{2}+y^{2}\right)\rho \left(x,y\right)\text{d}x\text{d}y\\ =\iint r2\rho \left(r,\theta \right)rdrd\theta \\ =\rho \int_{0}^{2\pi }\text{d}\theta \int_{0}^{M/\sqrt{K}}r^{3}\text{d}r=M^{2}/2\pi K. \end{array}$$

The total energy consumption of the network can be calculated as follows:

(6) $$\begin{array}{c} E_{\text{total}}=l\left(2{\text{NE}}_{\text{elec}}-kE_{\text{elec}}+k\varepsilon_{\text{amp}}d_{\text{CHtoBS}}^{2}+\left(N-k\right)\times \varepsilon_{\text{amp}}\times \frac{M^{2}}{2k\pi }\right). \end{array}$$

Substituting the calculated result of formula (5) into formula (4), calculating the derivative, the calculation result of the number of cluster heads *K* can be obtained, and at the same time, the derivative of the number of cluster heads *K* is equal to 0, and the calculation is obtained as follows:

(7) $$\begin{array}{c} E_{\text{elec}}-\frac{N\times M^{2}\times \varepsilon_{fs}}{\pi \times K^{2}}+\varepsilon_{\text{amp}}\times \left(d^{4}+d_{\text{CHtoBS}}^{4}\right)=0. \end{array}$$

Regarding the calculation of the optimal number of clusters *K* in formula (7), the first derivative of *K* can be calculated to obtain the calculation result of the optimal number of clusters *K* as follows:

(8) $$\begin{array}{c} K=M\times \sqrt{\frac{N\times \varepsilon_{fs}}{\pi }}\times \sqrt{\frac{1}{E_{\text{elec}}+\varepsilon_{\text{amp}}\times \left(d^{4}+d_{\text{CHtoBS}}^{4}\right)}}. \end{array}$$

From the calculation result of formula (8), the calculation result of the optimal number of clusters *K* is related to the number *N* of all sensor nodes in the monitoring area, the wireless communication distance *d* of the cluster head node's broadcast information, and the data transmission distance *d*_CHtoBS_ from the cluster head node to sink. The process of solving the optimal solution is a complex calculation process that requires comprehensive consideration of various factors, which is closely related to the application scenario and the user's purpose and needs. Generally, the calculation method of the optimal cluster number *K* value of HWSNs adopts the swarm intelligence optimization algorithm to optimize the solution [22].

In summary, the process of clustering data collection of HWSNs in this paper is as follows: in the stage of selecting cluster heads of HWSNs, an extreme learning machine neural network model is established through Sink, and the member nodes in the cluster are used as input vectors. The input parameters include the initial energy of the node, the number of neighbor nodes, and the distance between the heterogeneous node and the sink node. Through the adaptive learning of the extreme learning machine, the cluster head is selected as the output vector, which can reduce the energy consumption of the network. In the stage of optimizing the number of clusters, the method adopted by the classic SEP routing protocol is based on empirical values, which often has uncertainty, and the number of cluster heads in the network fluctuates greatly. Therefore, this paper uses the gray wolf algorithm to optimize the number of clusters of HWSNs. This method can effectively increase the number of data packets received by the sink node, improve the efficiency of data collection, and balance the energy consumption. Based on this, an energy-saving, efficient, and reliable clustering data collection strategy for HWSNs based on the online sequence extreme learning machine and gray wolf optimization algorithm is proposed in this paper, which can balance network energy consumption and prolong the network's lifetime.

## 4. Online Sequence Extreme Learning Machine and Gray Wolf Optimization Algorithm

### 4.1. Online Sequence Extreme Learning Machine

Online sequential extreme learning machine (OS-ELM) divides the training data into data blocks and trains in time sequence, which can effectively avoid repetition [23]. This algorithm mainly solves the problem that the extreme learning machine cannot dynamically process data in real time but can only retrain the old and new data together, which takes too long. The OS-ELM method can learn in batches and remove the trained data, which can reduce training time. It is a neural network learning and training process, which is mainly divided into two processes: initial learning and online learning [24].

In the OS-ELM algorithm, given *N* samples, Ω=(*x*_*i*_, *t*_*i*_), *i*=1,  2,  3,   …,  *N*_0_, *x*_*i*_ ∈ *R*^*m*^ is the input feature information, and *t*_*i*_ ∈ *R*^*n*^ is the idealized recognition output of the sample. The number of input layers is *n*, and the number of hidden layers is *L*. The network output can be expressed as follows:

(9) $$\begin{array}{c} f_{L}=\overset{L}{\underset{i=1}{\sum }}\beta_{i}\times g_{i}\left(a_{i},b_{i},x_{j}\right)=t_{i}. \end{array}$$

In the calculation and solution process, if the number of hidden layer nodes *L* of the extreme learning machine is equal to the number of input samples *N*, the output matrix will be a square matrix *H*, $\hat{\beta }=H^{-1}\times T$. If the number of *L* and *N* is not equal, that is, *L* << *N*, then they do not match. In this case, it is necessary to introduce least squares and Moore's generalized inverse to calculate *H* × *β*=*T* to obtain $\hat{\beta }=H^{+}\times T$, where *H*^+^ is the generalized inverse of the output matrix *H* [25].

In the online learning process, the training data are first divided in the form of data blocks, and the first sample set Ω_0_={(*x*_*i*_, *t*_*i*_)}_*i*=1_^*N*_0_^ is input, and the original output weight is solved according to $\hat{\beta }=H^{+}\times T$ as shown in formula (10). Among them, *P*_0_=*H*^*T*^*H*, *T*_0_=(*t*_1_, *t*_2_,…,*t*_*N*_0__)^*T*^, and add the data setΩ_1_={(*x*_*i*_, *t*_*i*_)}_*i*=*N*_0_+1_^*N*_0_+*N*_1_^, where the parameter *N*_1_ is the number of data set samples at the new time, and the corresponding weights are updated as shown in formula (11) [26].

(10) $$\begin{array}{c} \beta^{\left(0\right)}=P_{0}^{-1}H_{0}^{T}T_{0},\\ \beta^{\left(1\right)}=P_{1}^{-1}\left[\begin{array}{c} H\\ H_{1} \end{array}\right]\left[\begin{array}{c} T\\ T_{1} \end{array}\right]. \end{array}$$

Therefore, it can be calculated as follows:

(11) $$\begin{array}{c} \beta^{\left(1\right)}=\beta^{\left(0\right)}+P_{1}^{-1}H_{1}^{T}\left(T_{1}-H_{1}\beta^{\left(0\right)}\right). \end{array}$$

The data are constantly changing. When new data are added, the trained data should be removed to avoid repeated training. When the *K* + 1 batch of data is added, the output weight at this time is as follows:

(12) $$\begin{array}{c} \beta^{\left(K\right)}=\beta^{\left(K-1\right)}+P_{K+1}^{-1}H_{K+1}^{T}\left(T_{K+1}-H_{K+1}\beta^{\left(K\right)}\right). \end{array}$$

Parameter *P*_1_=*P*_*K*_+*H*_*K*+1_^*T*^*H*_*K*+1_. If the parameters *N*_*0*_ and *N* are consistent and equal, the OS-ELM algorithm is equivalent to the original ELM algorithm without any difference [27].

### 4.2. Gray Wolf Optimization Algorithm

Dr. Mirjalili and others proposed the gray wolf optimization algorithm (GWO) in 2014. The algorithm is inspired by the cooperation and hierarchy in the process of prey hunting by wolves and divides wolves into four levels [28]. The algorithm refers to the hunting division and food distribution of gray wolves in nature, takes artificial wolves as the main body, and uses a cooperative path search structure based on the division of responsibilities to abstract the engineering optimization solution process as a gray wolf hunting process [29].

Assuming that the wolf pack contains *N* individuals and searches for food in a *D*-dimensional space, the position of the *i*-th gray wolf that only captures food can be expressed by a mathematical formula as *X*_*i*_=(*X*_*i*_^1^, *X*_*i*_^2^,…, *X*_*i*_^*D*^), *i* = 1, 2,…, *N*, where the parameter *N* is the gray wolf population size. At the same time, it is defined that *α* (alpha) is the optimal solution to be found in the prey and so on, *β* (beta) is the second optimal solution, *δ* (delta) is the third optimal solution, and the remaining solution is *ω* (omega) [30]. The gray wolf individuals in the wolf pack are set as *X*, and the positions of the wolf individuals *α*, *β*, and *δ* in the wolf pack obtained by the iterative solution are *X*_*α*_, *X*_*β*_, and *X*_*δ*_ to update their respective positions. Their calculation formula is as follows:

(13) $$\begin{array}{c} X_{i,\alpha }^{d}\left(t+1\right)=X_{\alpha }^{d}\left(t\right)-A_{i,1}^{d}\left|C_{i,1}^{d}X_{\alpha }^{d}\left(t\right)-X_{i}^{d}\left(t\right)\right|,\\ X_{i,\beta }^{d}\left(t+1\right)=X_{\beta }^{d}\left(t\right)-A_{i,2}^{d}\left|C_{i,2}^{d}X_{\beta }^{d}\left(t\right)-X_{i}^{d}\left(t\right)\right|,\\ X_{i,\delta }^{d}\left(t+1\right)=X_{\delta }^{d}\left(t\right)-A_{i,3}^{d}\left|C_{i,3}^{d}X_{\delta }^{d}\left(t\right)-X_{i}^{d}\left(t\right)\right|. \end{array}$$

Here, the parameter *t* is the current iteration number; the parameters *X*_*α*_, *X*_*β*_, and *X*_*δ*_ are the positions of the current wolf population to capture prey; and *A*_*i*_^*d*^|*C*_*i*_^*d*^*X*_*α*_^*d*^(*t*) − *X*_*i*_^*d*^(*t*)| is the encircling step length, and the calculation formulas of the convergence factor *A*_*i*_^*d*^ and the swing factor *C*_*i*_^*d*^ are as follows:

(14) $$\begin{array}{c} A_{i}^{d}=2a\times {\text{rand}}_{1}-a,\\ C_{i}^{d}=2\;{\text{rand}}_{2}. \end{array}$$

The calculation formula of parameter *a* is as follows:

(15) $$\begin{array}{c} a=2-\frac{2t}{t_{\max}}. \end{array}$$

Here, the parameter *t* represents the current calculation times of the gray wolf population and *t*_max_ represents the iterative termination times of the gray wolf individual in the iterative calculation.

(16) $$\begin{array}{c} X_{i}^{d}\left(t+1\right)=\sum_{j=\alpha ,\beta ,\delta }w_{j}X_{i,j}^{d}\left(t+1\right). \end{array}$$

Here, *w*_*j*_(*j*=*α*, *β*, *δ*) represents the weight coefficient of gray wolf individual *α*, *β*, and *δ*; then it can be calculated as follows

(17) $$\begin{array}{c} w_{j}=\frac{f\left(X_{j}\left(t\right)\right)}{f\left(X_{\alpha }\left(t\right)\right)+f\left(X_{\beta }\left(t\right)\right)+f\left(X_{\delta }\left(t\right)\right)}. \end{array}$$

Here, *f*(*X*_*j*_(*t*)) represents the fitness value of the *j*-th wolf pack individual in the *t*-th generation.

The time complexity indirectly reflects the length of time the algorithm executes. In the GWO algorithm, it is assumed that the execution time required to initialize the parameters (under the condition that the population size is *N* and the spatial dimension is *n*) is *x*_1_, and the time to generate a uniform distribution is *x*_2_. The time required to find the fitness value is *f*(*n*), and then the time complexity of the initial stage of the GWO algorithm is as follows:

(18) $$\begin{array}{c} O\left(x_{1}+N\left(nx_{2}+f\left(n\right)\right)=O\left(n+f\left(n\right)\right)\right). \end{array}$$

Assuming that the execution time required for the iterative update of each dimension of the individual is the same, which is *x*_3_, the time for comparing the advantages and disadvantages and selecting the best after iteration is *x*_4_. Then the time complexity of the algorithm at this stage is as follows:

(19) $$\begin{array}{c} O\left(N\left(nx_{3}+f\left(n\right)\right)+x_{4}\right)=O\left(n+f\left(n\right)\right). \end{array}$$

Therefore, the total time complexity of the GWO algorithm is as follows:

(20) $$\begin{array}{c} T\left(n\right)=O\left(n+f\left(n\right)\right)+O\left(n+f\left(n\right)\right)=O\left(n+f\left(n\right)\right). \end{array}$$

In summary, the improved strategy of the GWO algorithm does not increase the time complexity of the algorithm solution compared to the other traditional optimization algorithms.

## 5. Design of Clustering Data Collection Algorithm for HWSNs

**Figure 2:**
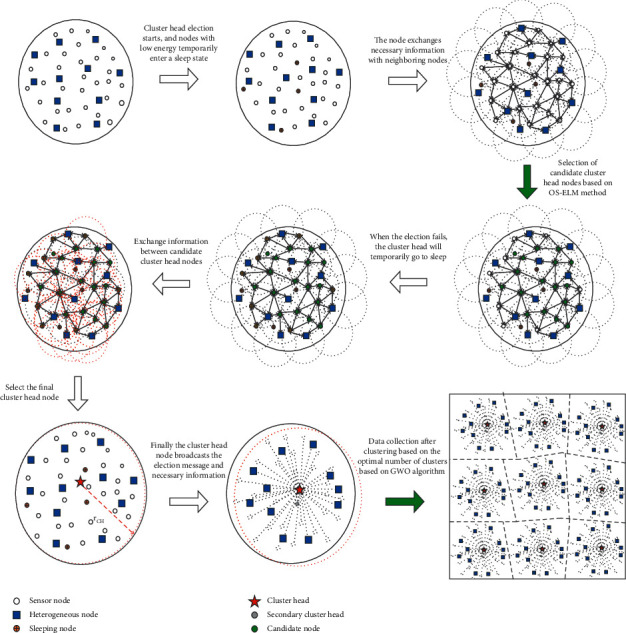
Clustering process of data collection algorithm.

The clustering data collection algorithm of HWSNs is improved on the basis of the clustering routing protocol (SEP) of classic HWSNs [31]. In the clustered data collection optimization strategy of HWSNs, data collection is divided into three stages: cluster head selection, cluster region formation, and data transmission between clusters. The heterogeneous sensor nodes in the network select the best cluster head node according to the neural network method of the online sequence extreme learning machine. Then a method for the optimal number of clusters of the gray wolf optimization algorithm is designed. After the number of clusters and the clustering area are completed, the data are transmitted between the clusters to the sink node. Finally, the data are sent to the server for data processing, and the entire data collection process is completed. The clustering data collection algorithm process of HWSNs is shown in Figure 2.

### 5.1. Clustering Stage

Since heterogeneous nodes are randomly deployed in a complex and changeable application environment, the specific location of the heterogeneous sensor nodes needs to be used to determine the partition. The methods for obtaining the location of specific sensor nodes mainly include the following three: (1) ranging, (2) nonrange positioning technology, and (3) node coordinate information.

### 5.2. Cluster Head Selection Stage

In the clustering data collection strategy of HWSNs, the key is the first step. The selection of cluster head nodes is related to the overall performance of network data collection. In this paper, the online sequence extreme learning machine neural network is used to select cluster heads, and the remaining energy of heterogeneous nodes, the number of neighbor nodes, and the distance between heterogeneous nodes and sink nodes are used as the input of the extreme learning machine neural network. All the information of the above network is transmitted to the sink node, and the online sequence extreme learning machine neural network is established in the sink for learning. The above heterogeneous sensor node information is used as the input vector of the online sequence extreme learning machine neural network. Through the continuous learning of the online sequence extreme learning machine, according to the information of the nodes in the monitoring area, the appropriate and optimal cluster head node is selected.

The design process of selecting the best cluster head is based on the adaptive learning method of the online sequence extreme learning machine:

**Step 1 .**

Set the parameter variables and HWSNs initialization parameters.

Assuming that *X*(*n*)=[*x*_1_(*n*), *x*_2_(*n*),…,*x*_*N*_(*n*)]^*T*^ is the input vector, the cluster heads are selected from these nodes in the monitoring area. The information of each node includes the remaining energy of the node, the number of neighbor nodes, and the distance between the cluster head node where it is located and the sink node. *ω*_*i*_^1^(*n*)=*ω*_*i*1_^1^(*n*), *ω*_*i*2_^1^(*n*),…,*ω*_*iN*_^1^(*n*)]^*T*^ is the weight coefficient of the forward subnetwork of ELM, *i*=1,2,3,…, *M*. The parameter *M* is the number of the heterogeneous nodes participating in the selection of cluster head nodes by improving ELM. *ω*_*kl*_^2^ is the weight of competition subnetwork of ELM, where the number 2 does not mean square but represents the second layer network of OS-ELM. *Y*(*n*)=[*y*_1_(*n*), *y*_2_(*n*),…, *y*_*l*_(*n*)] is the actual output vector of OS-ELM.

**Step 2 .**

Initialize.

For the forward network weight *ω*_*ij*_^1^, it is initialized with a small random value to make it meet the constraints

(21) $$\begin{array}{c} \overset{N}{\underset{j=1}{\sum }}\omega_{ij}^{1}=1,\;i=1,\;2,\;3,\;\ldots ,\;M. \end{array}$$

The setting of parameter *ω*_*kl*_^2^(*k*, *l*=1,2,3,…, *M*) is shown in the following formula (22):

(22) $$\begin{array}{c} \omega_{kl}^{2}=\left\{\begin{array}{c} 1,\;k=l\\ -\delta ,\;k\neq l \end{array}\right). \end{array}$$

Here, the parameters *k* and *l*, respectively, represent the current *k*-th and *l*-th sensor nodes in the heterogeneous sensor network.

In the learning process of the extreme learning machine, the output functions *f*_1_ and *f*_2_ of the neural network represent the functions; they are all linear relations, and the output expression formula is as follows:

(23) $$\begin{array}{c} y=\left\{\begin{array}{c} 0,\;x<\xi \\ x-\xi \;\xi ,\;<x<u_{c}\\ \text{constant }x,\;>u_{c} \end{array}\right). \end{array}$$

**Step 3 .**

Select the training sample *X*. After deploying the ordinary nodes and the heterogeneous sensor nodes in the monitoring area, all the nodes transmit their own information, including the remaining energy of the nodes, the number of neighbor nodes, and the distance between the cluster head node where they are located, to the sink node. The sink node uses the information of these nodes as the input vector of the neural network of the online sequence extreme learning machine, which is the current training sample.

**Step 4 .**

Calculate the output result of the forward subnetwork of the OS-ELM neuron as follows:

(24) $$\begin{array}{c} y_{k}\left(0\right)=V_{i}^{1}=f_{1}\left(\overset{N}{\underset{j=1}{\sum }}\omega_{ij}^{1}x_{j}\right),\;i=1,\;2,\;3,\;\ldots ,\;M. \end{array}$$

**Step 5 .**

Carry out the iterative process of calculating OS-ELM as follows:

(25) $$\begin{array}{c} y_{k}\left(n_{1}+1\right)=V_{i}^{1}=f_{2}\left(y_{k}\left(n_{1}\right)-\delta \underset{k\neq l}{\sum }y_{1}\right),\;k=1,\;2,\;3,\;\ldots ,\;M. \end{array}$$

**Step 6 .**

Obtain the output result of OS-ELM. After the output is completed, go to step 7, otherwise go to step 5 to continue the calculation, *n*_1_ = *n*_1_ + 1.

**Step 7 .**

Through calculation and comparison between neurons, select the largest neuron as the winning neuron, and then this neuron represents the sensor node selected as the cluster head node after the OS-ELM learning calculation.

**Step 8 .**

Update the sensor node currently selected as the cluster head node and update the weight of the current neuron as follows:

(26) $$\begin{array}{c} \omega_{i}^{1}\left(n_{1}+1\right)=\omega_{i}^{1}\left(n\right)+\eta \times \left[X_{i}\left(n\right)-\omega_{i}^{1}\left(n\right)\right]. \end{array}$$

**Step 9 .**

Determine whether the maximum number of iterations is reached; if not, return to step 3 to continue to calculate the comparison and select again. Otherwise, the calculation ends.

The OS-ELM learning method selected in this paper uses the unsupervised learning method to perform iterative calculation and training. When the training and calculation are completed, the cluster head nodes in the monitoring area are also selected.

### 5.3. The Formation Stage of Clusters

After selecting the cluster heads in the first stage, the second stage is the determination of the number of clusters. The number of clusters also determines the performance of the network. Too many clusters will cause frequent communication between cluster heads and sink nodes to generate greater energy consumption. If the number of clusters is too small, multihop transmission between nodes will increase energy consumption. Considering various factors, an optimal cluster number method for HWSNs based on the gray wolf optimization algorithm is proposed in this paper. This method avoids too many or too few clusters of HWSNs, balances the number of clusters in the network, balances network energy consumption, improves the efficiency of data collection, and reduces the delay of data transmission.

### 5.4. Data Transfer between Clusters

After the clustering is formed, it enters the data transmission stage. When the heterogeneous sensor nodes are deployed, data collection starts, and the collected data is sent to the cluster head node within a specified time. The cluster head node performs data fusion and then sends the data to the sink node. After the data transmission is completed, the node enters the dormant state, which reduces the energy consumption of the network.

### 5.5. Stable Operation Stage

In the set time, the cluster head node receives the data and then sends it to the sink node to complete the routing transmission between clusters. In the data collection process, the cluster head is selected according to the set threshold. If a node has been selected as the cluster head, it will not participate in the selection in the next round. After multiple rounds of data collection, the clustered data collection of HWSNs in the monitoring area is finally completed.

## 6. Simulation Comparison and Result Analysis

### 6.1. Simulation Environment Settings

In the construction of the simulation environment, we use MATLAB 2017a software to simulate and verify the clustering data collection strategy of the proposed online sequence extreme learning machine and gray wolf optimization algorithm. Common nodes and heterogeneous nodes are randomly deployed in a 500 × 500 m^2^ square monitoring area. The total number of sensor nodes is set to 200, and the initial energy is set to 1 J. Common nodes and heterogeneous nodes send 10 data packets every 1 minute. The length of the packet is 4,000 bits, sent to the cluster head, and forwarded to the sink after fusion processing. The ELM parameter setting lies in its network structure, the number of hidden layers, and the kernel function adopted by the hidden layers. The initial number of hidden layer nodes is set to 10; gradually increased with a period of 20, until reaching the value 300; and chosen hard limit as the hidden layer activation function. The gray wolf optimization algorithm parameter settings in the optimal number of clusters: the gray wolf population is 50; the number of iterations is 400; and the wolf group control parameters are *α*1 = 0.9 and *α*2 = 0.4.

In order to reflect the performance of the algorithm in this paper, this paper proposes a data collection method (OS-ELM + GWO) based on the cluster head selection of the online sequence extreme learning machine and the gray wolf optimization of the optimal number of clusters. Experiments were compared with SEP data collection protocol, ELM-based cluster head selection method, ELM-based cluster head selection, and PSO algorithm optimization optimal cluster head number data collection method (ELM + PSO) to verify the superior performance of the proposed algorithm.

### 6.2. Simulation Comparison and Analysis

There are many indicators that affect the network performance of HWSNs. This paper mainly conducts experiments from the clustering effect of HWSNs, the total energy consumption of the network, the number of cluster head nodes, the energy consumption of the cluster head nodes, the number of data packets received by the sink, network load balancing, transmission delay, and reliability. These indicators can reflect the superior performance of the algorithm proposed in this paper.

In order to verify the superior performance of the gray wolf algorithm, we compare it with the three algorithms of particle swarm optimization (PSO), ant colony algorithm (ACO), and the new monarch butterfly algorithm (MBO). We solve the optimal values of the four functions, and the expressions of the four functions are shown in Table 1.

**Figure 3:**
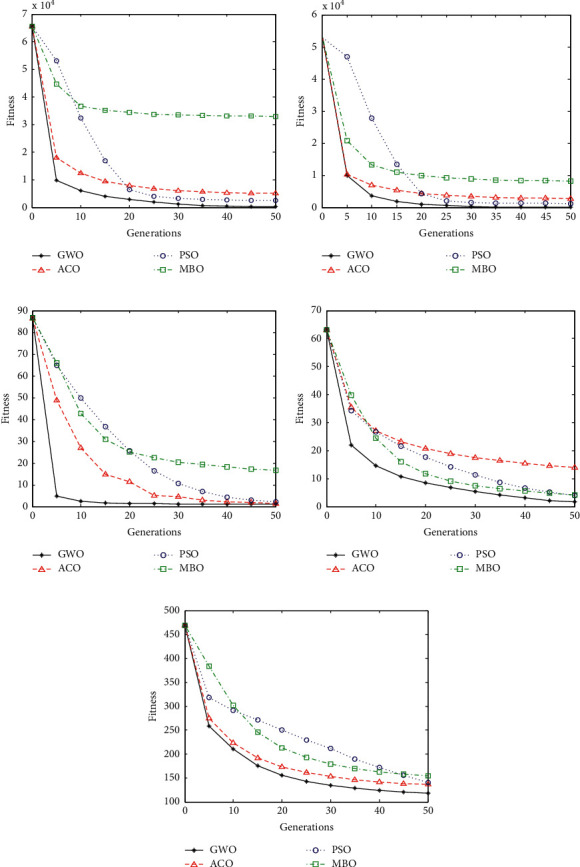
Comparison of convergence curves for function optimization: (a) F1, (b) F2, (c) F3, (d) F4, and (e) F5.

The iterative solution process of the optimal solution of the five functions is shown in Figure 3.

From the process of finding the optimal solution of the five functions in Figure 3, it can be seen that with the progress of iteration, the gray wolf algorithm has the best performance, the fastest search speed, and the best optimal solution.

#### 6.2.1. Comparison of Network Clustering Effects

**Figure 4:**
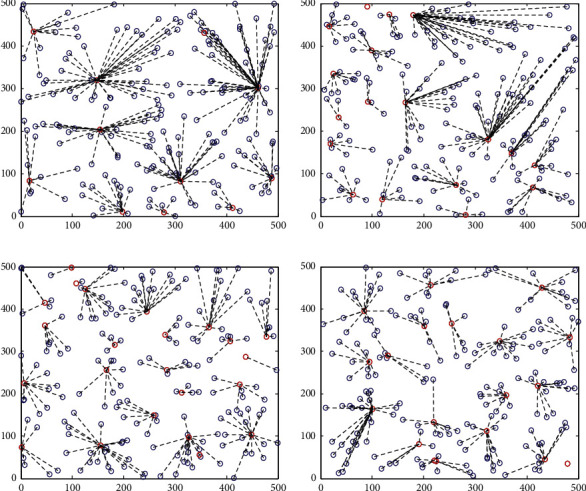
Schematic diagram of network clustering of HWSNs: (a) SEP, (b) ELM, (c) ELM + PSO, and (d) OSELM + GWO.

Whether the network clustering is balanced or not directly affects the network energy consumption and network life. If the network clustering is large, the nodes need to consume more energy to transmit data farther. If the clustered area is small, multihop data transmission between nodes will extend the data transmission time. The clustering effect of HWSNs is shown in Figure 4.

From the four clustering methods in Figure 3, it can be seen that the data collection and clustering method of the SEP routing protocol has the largest clustering area, the distribution between clusters is not uniform, and the number of member nodes in each cluster is different. The ELM-based data collection strategy has a larger cluster size, and the number of clusters is more uniform. The data collection method based on ELM + PSO has a more uniform clustering area, but the clustering area is larger in individual places. There are also a few clustering areas where there is only one cluster head node and cluster member node, and the clustered data and size of the nodes are relatively uniform. The OSELM + GWO data collection method proposed in this paper has a more uniform clustering area, a more uniform number of cluster member nodes, and a more uniform number of clusters. In general, the OSELM + GWO data collection method proposed in this paper has an ideal clustering effect.

#### 6.2.2. Energy Consumption Comparison

**Figure 5:**
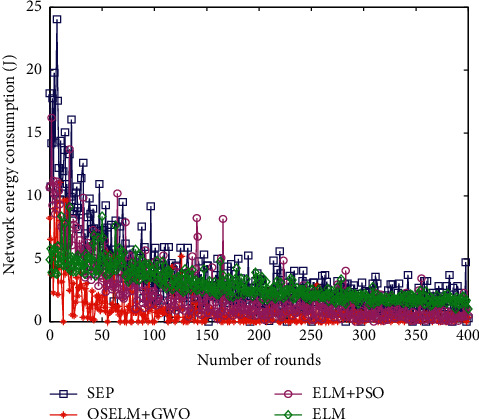
Comparison of energy consumption.

Network energy consumption directly affects the performance and work efficiency of heterogeneous wireless sensor networks. The proposed method is compared with the SEP data collection protocol, the ELM-based cluster head selection method, the ELM-based cluster head selection, and the PSO algorithm to optimize the optimal number of cluster heads data collection method (ELM + PSO). The energy consumption curve of the four algorithms is shown in Figure 5.

It can be seen from the energy consumption change curve that the classical SEP data collection method randomly selects cluster heads, and the energy consumption is the largest. The data collection algorithm based on the ELM method consumes a lot of energy, and the data collection method based on ELM + PSO has less energy consumption. The OSELM + GWO data collection method proposed in this paper has the least energy consumption. In terms of 200 polling times, the OSELM + GWO data collection method proposed in this paper saves 43.7% of the energy consumption of the SEP routing protocol. Compared with the optimal cluster head data collection algorithm based on the ELM method, the energy consumption is saved by 31.8%, and the energy consumption is saved by 20.5% compared with the ELM + PSO method. On the whole, the OSELM + GWO data collection method proposed in this paper has the least energy consumption.

**Figure 6:**
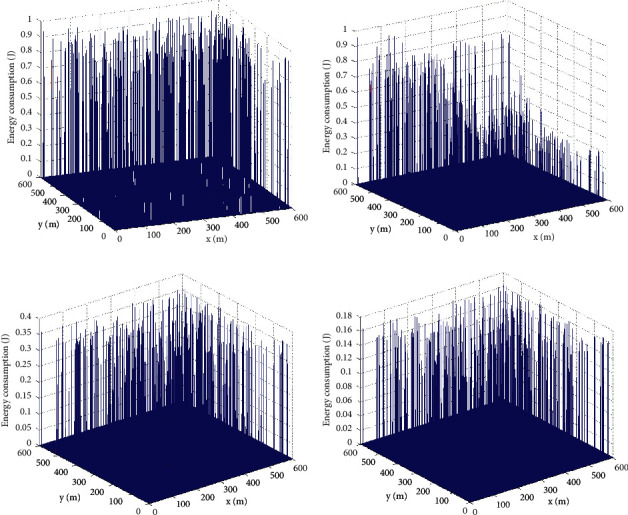
Comparison of energy consumption of three dimensional: (a) SEP, (b) ELM, (c) ELM + PSO, and (d) OSELM + GWO.

The comparison of the energy consumption of the three-dimensional network of the four algorithms is shown in Figure 6.

#### 6.2.3. Comparison of the Number of Surviving Nodes

**Figure 7:**
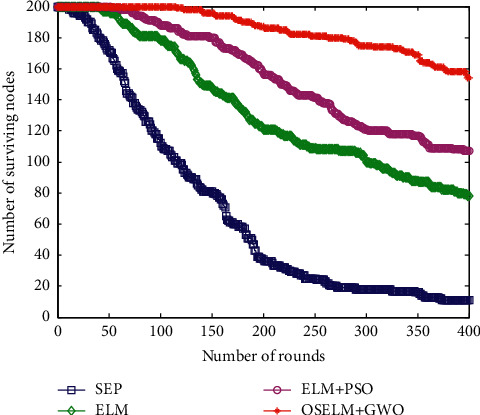
Comparison of the number of the surviving nodes.

The number of surviving nodes in the network reflects the life of the network, which is one of the purposes of network performance optimization. The more surviving nodes, the longer the working time of the network. The comparison of the number of surviving nodes in the network of the four algorithms is shown in Figure 7.

With the increase of simulation polling times, the survival of HWSNs sensor nodes of the four algorithms gradually decreases. This is mainly because the energy consumption of the network is gradually increasing with the increase of simulation time. Among them, the data collection method of the SEP routing protocol has the least number of surviving nodes, and the number of surviving nodes has the largest decrease. The data collection method based on the ELM method has fewer surviving nodes, and the data collection method based on ELM + PSO has a smaller decline in the number of surviving nodes. The method proposed in this paper has the largest number of surviving nodes, the smallest decline, and the longest life cycle of nodes in the network.

#### 6.2.4. Comparison of the Number of Cluster Heads

**Figure 8:**
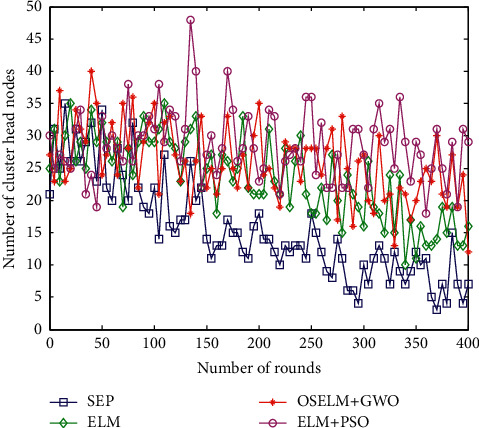
Comparison of the number of the cluster head nodes.

In the HWSNs clustered data collection strategy, the number of clusters affects the final work efficiency of data collection. If the number of clusters of HWSNs is small, the cluster area in the monitoring area is larger; the node transmission distance is longer; and the long-distance transmission causes an increase in energy consumption. If the number of clusters is large, the nodes are scattered, and multihop data transmission causes data delay, which affects the efficiency of data collection. Therefore, it is necessary to balance the number of cluster head nodes and select the number of cluster head nodes reasonably. The comparison of the number of cluster heads of the four algorithms is shown in Figure 8.

The number of cluster heads of the four algorithms HWSNs in Figure 8 is relatively balanced and fluctuates little with the increase of the number of polls. The number of cluster head nodes of the SEP data collection strategy dropped from 25 to about 8, with the largest decline and the largest fluctuation. The data collection strategy of the ELM method has a number of cluster head nodes ranging from 35 to 15, and the decline is also relatively large. The data collection strategy based on ELM + PSO has little fluctuation and is relatively balanced, and the number of cluster heads in the algorithm proposed in this paper is not much different, and the number of clusters is also not much different. This paper proposes the OSELM + GWO data collection method to select the number of cluster head nodes to fluctuate smoothly, keeping it between 20 and 35, and the number of selected cluster heads is more reasonable.

#### 6.2.5. Comparison of Energy Consumption of Cluster Head Nodes

**Figure 9:**
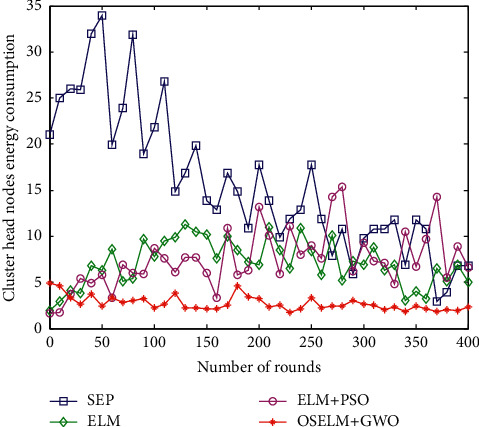
Comparison of energy consumption of cluster head nodes.

The energy consumption of the cluster head node also reflects the performance of HWSNs. The lower the energy consumption of the cluster head node, the better the network clustering effect. Otherwise, the energy consumption of the cluster head node will be exhausted in advance, leading to link failure and network paralysis. The change curve of cluster head node energy consumption is shown in Figure 9.

The SEP algorithm in Figure 9 is quite different from the other three methods. This is mainly because the latter three algorithms use the extreme learning machine method to select cluster heads, while the SEP algorithm selects randomly, which causes the energy consumption of the cluster head nodes to vary greatly and fluctuates the most. The method proposed in this paper has the least energy consumption and the smallest change range. The OSELM + GWO proposed in this paper is much better than the other three methods in selecting cluster heads and the number of cluster heads.

#### 6.2.6. The Number of Packets Received by Sink

**Figure 10:**
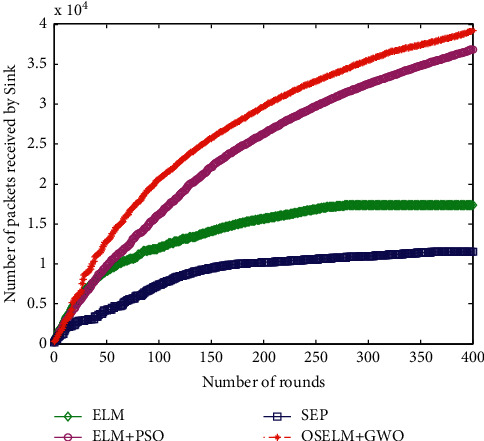
Comparison of the number of packets received by Sink.

The number of data packets received by sink is a direct manifestation of the final effect of HWSNs data collection. The more data packets are received; it indicates that the optimization strategy of the proposed data collection method is optimal.  Figure 10 shows the comparison result of data packets received by the sink node.

It can be seen from Figure 10 that the number of data packets received by the sink node for these four algorithms is very different. Among them, the SEP routing protocol method and the ELM-based data collection strategy sink receive less data packets, while the latter two receive more data packets. The latter two algorithms increase the optimization of the number of clusters compared with the previous two algorithms, which shows that the number of clusters in the second step has a great impact on the amount of data packets received by the sink node. The selection of the cluster head has little effect on the number of packets received by the sink node. From the comparison of the performance of the four algorithms, the number of data packets received by the sink node based on the ELM + PSO and OSELM + GWO data collection methods has increased significantly, and the sink receives the largest number of data packets, and the method is the best.

#### 6.2.7. Network Load Balancing Analysis

Network load balance affects network energy consumption and life cycle, which is an important indicator of HWSNs data collection. The load balancing factor (*L*_*LBF*_) of HWSNs refers to the reciprocal of the variance of all nodes in the monitoring area. The larger the value, the better the network load balance. The specific calculation formula is shown in the equation:

(27) $$\begin{array}{c} L_{\text{LBF}}=\frac{n_{c}}{\overset{n_{c}}{\underset{i=1}{\sum }}{\left(x_{i}-u\right)}^{2}}. \end{array}$$

**Figure 11:**
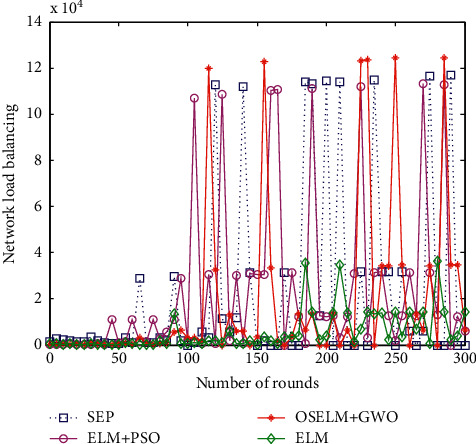
Comparison of network load balancing.

Here, parameter *n*_*c*_ represents the total number of sensor nodes deployed in the monitoring area, parameter *x*_*i*_ is the number of cluster member nodes added to the *i*-th clustered area, and parameter *u* is the average number of cluster member nodes in all clusters. After several polls, the load balance has not changed much. We show the network load balance comparison curve of the four algorithms for the previous 300 polls as shown in Figure 11.

It can be seen from the comparison in Figure 9 that the load balance of the classic SEP routing protocol network is the worst, and the OSELM + GWO data collection strategy proposed in this paper has a better load balance. The load balance based on the ELM data collection method and the ELM + PSO method lies between the two. This load balancing is mainly reflected in 100–300 rounds of iterations and mainly reflects the impact on network load balance in the second stage of data collection.

#### 6.2.8. Comparison of Network Transmission Delay

The transmission delay is calculated by the time the sink node receives the data packet, the shorter the better. Assume that the terminal node sends a data packet to the sink at the time *T*_*s*_, and the sink node receives the data packet at the time *T*_*r*_. The data transmission delay formula is as follows:

(28) $$\begin{array}{c} T_{\text{trans}}=\frac{1}{N_{r}}\overset{N_{r}}{\underset{i=1}{\sum }}\left(T_{ri}-T_{si}\right). \end{array}$$

**Figure 12:**
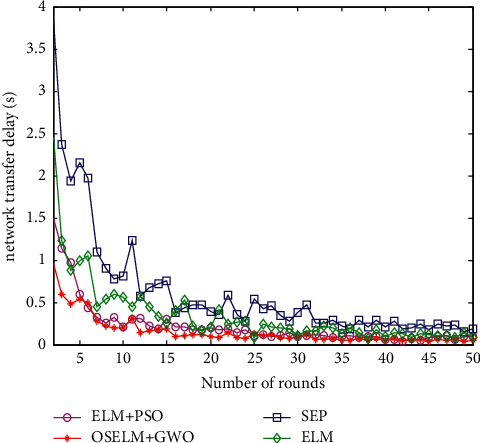
Comparison of the transmission delay.

Here, the parameter *N*_*r*_ is the total number of successfully received data packets. The algorithm has little change in the network transmission delay in the back, so we give the network delay comparison of the first 50 polling times as shown in Figure 12.

From the perspective of the overall network delay in Figure 12, the average transmission delay of the SEP routing protocol network is the largest, around 1.1 s. The transmission delay based on the ELM data collection method is relatively large, around 0.8 s, and the transmission delay based on the ELM + PSO data collection strategy is relatively small, around 0.5 s. The transmission delay of the proposed OSELM + GWO data collection strategy network is the smallest, about 0.3 s on average. It can be seen that the data collection method proposed in this paper has the shortest transmission delay.

#### 6.2.9. Comparison of Network Reliability

The current network reliability is an important indicator of network performance. There are many parameters that affect the network reliability *R*_net_, which can be calculated through the connectivity *I*_1_ between the end nodes, the network connectivity rate *I*_2_, and the network capacity *I*_3_. The formula for reliability is as follows:

(29) $$\begin{array}{c} R_{\text{net}}=0.1667I_{1}+0.5I_{2}+0.3333I_{3}. \end{array}$$

**Figure 13:**
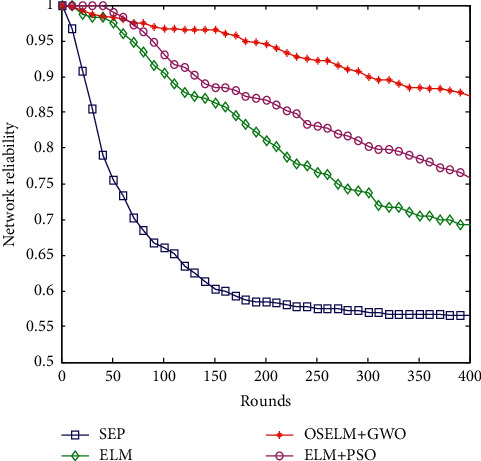
Comparison of network reliability.

The reliability matrix is calculated according to the distance between nodes, combined with Monte Carlo analysis, the connectivity *I*_1_ between all end nodes can be calculated after 400 polls. The network capacity *I*_3_ represents the survival probability of all nodes, and its value is the ratio of the number of all surviving nodes to the total number of nodes in the network. The network reliability values of the four algorithms are calculated, and the reliability comparison is shown in Figure 13.

In Figure 13, as the number of polls gradually increases, the network reliability of the four algorithms is decreasing, but the magnitude of the decrease is different. This is mainly due to the increased energy consumption of nodes, leading to the death of some nodes, affecting data transmission and affecting network performance. It can also be seen that the OSELM + GWO data collection method proposed in this paper has the smallest decrease in network reliability and the highest network reliability. Taking the network reliability of 400 polling times as an example, the data collection algorithm of the SEP routing protocol has the lowest network reliability, with a value of 0.57. The network reliability of ELM-based data collection algorithms is low, with a value of 0.69. The network reliability based on the ELM + PSO data collection algorithm is relatively high, and its value is 0.77. The OSELM + GWO data collection method proposed in this paper has the highest network reliability, with a value of 0.88.

In summary, the OSELM + GWO data collection method proposed in this paper is compared with the classic SEP routing protocol, the method of selecting cluster heads based on ELM, the selection of cluster heads based on ELM, and the PSO algorithm to optimize the number of cluster heads. The OSELM + GWO data collection strategy proposed in this paper receives the most data packets, the highest data collection efficiency, the longest lifespan, the lowest energy consumption, the highest reliability, and the best performance.

## 7. Conclusion

This paper proposed an energy-saving, efficient, and reliable clustering data collection method for HWSNs based on online sequence extreme learning machine and gray wolf optimization algorithm. The online sequence extreme learning machine adaptively learns to select the best cluster head, avoiding improper selection and frequent selection of cluster heads, which could cause energy waste. At the same time, a gray wolf optimization algorithm HWSNs optimal clustering number method was designed to avoid too many or too few cluster heads, reduce data transmission delay, and balance network energy consumption. The algorithm could balance the clustering effect, reduce network energy consumption, and increase the number of surviving nodes under the condition of ensuring the requirements of data delay. On the premise of increasing the data packets received by the sink node, the network lifetime was prolonged.

This paper currently focuses on the research on data collection of heterogeneous wireless sensor networks and then plans to carry out the research on heterogeneous mobile wireless sensor networks, as well as the use of the latest algorithms to study the optimal number of clusters in heterogeneous sensor networks.

## Figures and Tables

**Table 1:** The basic information of the test function.

Function	Formula	Dimension	Bounds	Optimum
Sphere	$F1=\sum_{i=1}^{d}x_{i}^{2}$	30	_[−100, 100]_	0
Step	$F2=\sum_{i=1}^{d}{\left(\left|x_{i}+\text{0}\text{.}5\right|\right)}^{2}$	30	_[−100, 100]_	0
Quartic	$F3=\sum_{i=1}^{d}ix_{i}^{4}+\text{rand}\left(0,1\right)$	30	_[−1.28, 1.28]_	0
Alpine	$F4=\sum_{i=1}^{d}\left|x_{i}\text{sin}\left(x_{i}\right)+0.1x_{i}\right|$	30	_[−10, 100]_	0
Rastrigin	$F5=10\;d+\sum_{i=1}^{d}\left[x_{i}^{2}-10\text{cos}\left(2\pi x_{i}\right)\right]$	30	_[−5.12, 5.12]_	0

## Data Availability

The data used to support the findings of this study are available from the corresponding author upon request.
